# Supplementary material for: Unilateral Agenesis of the Upper Permanent Lateral Incisors in Growing Patients: Gap Closure or Gap Opening? A Systematic Review
Source: Int Dent J. 2025 Jul 3;75(4):100815. doi: 10.1016/j.identj.2025.03.024 (PMC12271859; doi:10.1016/j.identj.2025.03.024)
Supplement: Supplementary file 1 [file mmc1.pdf]

Plagiarism Checker X - Report

Originality Assessment

12%

Overall Similarity

Date: nov 20, 2024 (02:54)
Matches: 644 / 5470 words
Sources: 33

Remarks: Moderate similarity detected, consider enhancing the document if necessary.

Verify Report:
Scan this QR Code

1.1 Definition of Agenesis

The term 'agenesis' derives from two Greek components: the prefix 'alpha privative' (the letter "a," denoting absence) and "genesis" (meaning "birth" or "generation"). Thus, "agenesis" refers to the absence of development. "Dental agenesis," or congenital absence of teeth, describes teeth that teeth that fail to develop and are congenitally missing.

This condition can affect both primary (baby) teeth—though this is rare—and permanent teeth (1). One or more teeth are not visible clinically or radiographically at the age they should be present. For primary teeth, it is important to rule out previous extractions or exfoliation. In recent years, considerable research has focused on dental agenesis due to its high prevalence and the significant aesthetic and functional issues it creates, necessitating treatment.

Dental agenesis falls under "defects in tooth number" in the classification of dental anomalies (2):

I. Numerical anomalies

II. Size anomalies

III. Shape anomalies

IV. Positional anomalies

Among the classifications, the system proposed by Malavez et al. (1953) is widely used for its clarity and simplicity (3):

Anodontia: Total absence of both primary and permanent teeth, a very rare condition.

Oligodontia: Multiple agenesis, involving at least half of the teeth in a dental arch.

Hypodontia: 17 Agenesis of fewer than half of the teeth. Maxillary lateral incisor unilateral agenesis (UMLIA) is a type of hypodontia.

Beyond the number of missing teeth, it can be useful to classify agenesis by the affected area of the dental arches—anterior, middle, posterior, or mixed. A topographic classification helps to clarify the specific problems associated with agenesis. 5 In the anterior region, agenesis leads to aesthetic and occlusal issues (e.g., missing upper lateral incisors), while in the middle and posterior regions, it primarily causes functional problems

(3).

1.2 Growth

While "growth" and "development" are often confused, they have distinct meanings. Growth is an anatomical process, while development is physiological (5).

For females and males, adolescent growth typically begins at ages 9.5 and 10.5, respectively. During early adolescence, growth accelerates until it reaches a peak, after which it gradually slows. If a patient's skeletal age indicates they are in the peak phase, about three more years of significant craniofacial development can be expected. In females, the onset of menstruation (menarche) is a reliable indicator of sexual maturity, which also triggers a sudden growth spurt (4).

Dentists, especially orthodontists, play a crucial role in guiding facial growth and development. Accurate assessment of skeletal age allows clinicians to optimize the timing of orthodontic or orthopedic treatments, leveraging the peak growth phase for the best therapeutic outcomes. Procedures like surgical dental implants must also consider the patient's growth stage, as implants should only be placed after cranial growth is complete. Radiographs are essential in determining if bone growth has fully ceased (5).

There are various methods for evaluating bone growth, with hand and wrist radiographs being the most commonly used by orthodontists. This method, while reliable, requires additional imaging beyond those already needed for orthodontic treatment planning (6). A more recent method involves analyzing the shape of cervical vertebrae (C2, C3, and C4) visible in lateral cephalometric radiographs, which are routinely used in orthodontic assessments. The vertebral body shape and radiolucency provide a snapshot of the degree of ossification, organized into six stages. CS1 to CS4 are key stages for determining optimal timing for orthopedic and orthodontic interventions, while CS5 and CS6 mark the end of growth (7). Magnetic resonance imaging (MRI) can also be useful for growth studies, offering 3D imaging without radiation, although it better visualizes soft tissues than hard tissues (5).

1.3 Epidemiology

[12] Dental agenesis is one of the most common developmental anomalies, frequently affecting maxillary lateral incisors. Hypodontia in primary dentition is rare, with a prevalence between 0.1% and 0.7%. When it does occur, it typically affects the incisors, particularly the upper lateral incisors. If a primary tooth is missing, many studies report a nearly 100% chance that its permanent successor will also be absent, as the permanent tooth develops from the dental lamina of the primary tooth (3).

There is also a strong association between tooth fusion in primary dentition and agenesis [5] in the permanent dentition. Specifically, fusion of a temporary lateral incisor and canine often predicts agenesis of the permanent lateral incisor. Cases of fusion between a temporary lateral and central incisor, followed by permanent lateral agenesis, have also been reported (8).

The most commonly missing permanent teeth vary depending on the population studied. Excluding third molars, lower second premolars (47.5%), upper lateral incisors (30.5%), and upper second premolars (23.5%) are frequently absent, though these percentages differ by region. There is a general trend towards missing the most distal teeth in each group: lateral incisors within the incisor group, second premolars in the premolar group, and third molars among the molars (3).

A meta-analysis by Rakhshan et al. (2013) found that the prevalence of dental agenesis ranges from 0.1% to 16.2%, with most studies reporting around 7% (9).

Agenesis may affect a single tooth or multiple teeth, and can occur symmetrically or asymmetrically in one or both arches. Hypodontia is relatively common, while the absence of more teeth (e.g., anodontia) is rare. Some studies indicate that 43% of agenesis cases involve [17] a single missing tooth, 40% involve two, and 15% involve three (3).

The absence of third molars is considered an evolutionary adaptation rather than a developmental defect. Proffit et al. (2001) suggested that evolutionary forces have reduced the number and size of teeth, as well as jaw size, in contemporary humans (10).

As [4] for maxillary lateral incisor agenesis in permanent dentition, its global prevalence ranges between 0.8% and 4.25%, according to studies by Teresa Pinho in 2011 (8).

In summary:

Permanent dentition is more commonly affected than primary dentition (3).

In Europeans, mandibular second premolars are most frequently missing, followed by [1] maxillary lateral incisors (with prevalence decreasing from northern to southern Europe). In American, Malaysian, and Israeli populations, maxillary lateral incisors are most frequently absent (8).

Females are more commonly affected than males, with a ratio of 3:2 (3).

In unilateral agenesis of the upper lateral incisors, the right side is more commonly affected than the left. However, bilateral absence is more common [8] in the case of maxillary lateral incisor agenesis (11).

1.4 Etiology

Dental agenesis can occur in both completely healthy individuals and in those with complex syndromes or congenital malformations, with non-syndromic hypodontia being the most prevalent form. According to the literature, multiple causes can lead to dental agenesis, including that of the maxillary lateral incisor. However, the etiopathogenesis of hypodontia remains poorly understood and is likely the result of numerous factors and their potential interactions.

1.4.1 Genetic Causes

Agenesis can result from developmental disturbances during the initiation or proliferation phases of tooth formation. In recent years, significant advances in genetic and molecular biology research on odontogenesis have aimed to identify the mutations responsible for dental agenesis.

[29] Odontogenesis is a complex process regulated by reciprocal epithelial-mesenchymal interactions, under genetic control, which determines tooth position, number, shape, and size. Any mutations affecting genes involved in these processes can lead to abnormal tooth development, including agenesis. Notably, mutations in genes such as [12] MSX1, PAX9, and AXIN2 have been associated with dental agenesis in humans.

Genetic factors are further supported by the observation of familial aggregation in cases [4] of maxillary lateral incisor agenesis. For example, Pinho et al. studied the risk of lateral incisor agenesis among first-degree relatives of patients in populations from Portugal, Sweden, Utah, and Israel. They found that first-degree relatives had a significantly higher risk—15, 16, 12, and 5 times greater, respectively—of experiencing the same type of agenesis compared to the general population.

1.4.2 General Causes

Agenesis can also be attributed to systemic conditions such as rickets, congenital syphilis, maternal nutritional deficiencies during pregnancy, or other significant maternal health conditions during the first month of gestation.

1.4.3 Local Causes

Local factors such as chemotherapy agents and radiation therapy can irreversibly impact tooth development. The effects depend on the patient's age and dose. Trauma, osteomyelitis, or maxillofacial surgery can also damage formed tooth germs, leading to their loss, which is considered secondary rather than true agenesis.

1.4.4 Syndromic Causes

Several genetic conditions are associated with dental agenesis. In some cases, [6] only one or a few teeth are missing, while in others, multiple teeth are absent. For instance, patients with Down syndrome frequently exhibit higher rates of dental agenesis, particularly involving permanent teeth, compared to other syndromes. Down syndrome, caused by the triplication of chromosome 21, leads to intellectual disabilities and physical deformities, with 65% of patients experiencing [5] the absence of one or more teeth, most commonly the maxillary lateral incisors. These patients also often present various occlusal abnormalities, necessitating special care to ensure proper oral function and to prevent dental pathologies.

Cleft palate patients also commonly exhibit dental agenesis. [6] The development of the alveolar process in these patients is compromised, often leading to dental anomalies, particularly agenesis of the maxillary lateral incisors.

Ectodermal dysplasias are another group of genetic disorders associated with dental agenesis. These conditions affect not only the teeth but also 30 the skin, sweat glands, hair, and nails. Some patients with ectodermal dysplasia may also have a cleft palate. The most common types of ectodermal dysplasia are hypohidrotic and hydrotic. Hypohidrotic ectodermal dysplasia (Christ-Siemens-Touraine syndrome) 6 is characterized by a triad of hypotrichosis (abnormalities of skin, hair, and nails), hypodontia, particularly affecting the maxillary lateral incisors, and hypohidrosis (reduced sweating due to absence of sweat glands). These patients often present with other dental anomalies, such as altered tooth shape, delayed eruption, and tooth impaction.

1.5 Clinical Characteristics

Dental agenesis is frequently associated with other dental anomalies, such as microdontia or peg-shaped maxillary lateral incisors (especially in cases of unilateral agenesis), taurodontism, transpositions, supernumerary teeth, ectopic eruption, and retained primary teeth.

Celikoglu et al. highlighted that 5 the most common dental anomalies associated with maxillary lateral incisor agenesis are:

Ectopic eruption of the maxillary canine

Reduced or conoid contralateral incisor

Hypodontia of other teeth, excluding third molars

Dilaceration

Impaction of the maxillary canine

Maxillary lateral incisor-canine transposition

Maxillary canine transmigration

Supernumerary teeth (although rare)

Delayed 6 dental development

The most commonly observed anomalies are ectopic eruption of the maxillary canines and 11 the presence of a reduced or conoid contralateral lateral incisor. The lateral incisor’s root plays a guiding role for 18 the eruption of the maxillary canine, and when it is absent

or altered, the canine may erupt ectopically or become impacted.

There is a direct relationship between tooth size reduction or microdontia [19] and the number of missing teeth, with more severe reductions in mesiodistal width correlating with increased numbers of absent teeth.

1.6 Diagnosis

The diagnosis of dental agenesis, including [4] maxillary lateral incisor agenesis, can often be an incidental finding during a routine dental examination. The anamnesis is crucial for evaluating any history of extractions or trauma, as well as identifying any familial genetic predispositions. The diagnosis is both clinical and radiological, and hypodontia can be suspected based on clinical signs such as persistent retention of a primary lateral incisor beyond the expected eruption time of its permanent successor, an asymmetry in the loss [6] of primary teeth, or midline deviation toward the side of agenesis in cases of unilateral absence.

Radiographic examination is essential for confirming the diagnosis, and advanced [9] imaging techniques such as cone-beam computed tomography (CBCT) offer detailed three-dimensional analysis, particularly useful when planning treatments like dental implants.

1.7 Treatment

In young patients, the absence of an anterior tooth, such as the maxillary lateral incisor, can have a significant impact on their quality of life. Managing lateral incisor agenesis requires a multidisciplinary approach involving orthodontists, periodontists, implantologists, prosthodontists, and restorative dentists, ensuring effective communication among professionals to achieve optimal outcomes.

Two main treatment options have been described in the literature:

Space opening followed by placement of an implant, or [1] the use of a fixed or removable prosthesis to replace the missing tooth

Space closure, with the maxillary canine being aesthetically camouflaged to substitute the lateral incisor, and the first premolar reshaped to resemble the canine.

Selecting between these options depends on various factors, including the patient's age, incisor protrusion, facial patterns, dental arch configuration, tooth shape, gingival contour, aesthetic considerations, and other factors affecting treatment outcomes.

2. MATERIALS AND METHODS

This systematic review was conducted following the guidelines of the Preferred Reporting Items for Systematic Reviews and Meta-Analyses (PRISMA) statement.

2.1 Eligibility Criteria

PICO Question:

Patients/Problems: Growing [1] patients with unilateral agenesis of the permanent maxillary lateral incisor.

Intervention: Space opening followed by implant placement.

Comparison: Space closure and canine reshaping.

Outcomes: Aesthetic and periodontal results obtained from different therapeutic approaches.

2.2 Research Question:

Which treatment yields the best aesthetic and periodontal outcomes in growing [1] patients with unilateral agenesis of the maxillary lateral incisor?

All studies that evaluated and compared outcomes of space opening versus space closure in growing patients with unilateral agenesis of the maxillary lateral incisor were included. Studies involving patients with unilateral and bilateral agenesis of the permanent maxillary lateral incisor were considered.

For the space closure approach, studies involving patients treated with both fixed and removable orthodontic appliances were included. Additionally, research using newer

technologies, such as Invisalign clear aligners, was considered. Studies focusing on space opening included those addressing restorative dental treatments, implantology, and both fixed and removable prosthetics.

Further inclusion criteria were based on the language and publication date. Articles published in English, Spanish, or Italian between 2012 and 2024 were included, ensuring a focus on recent advancements in treatment techniques. Only studies involving human subjects were considered.

Exclusion criteria included studies examining space closure or opening in adult patients or those with genetic syndromes. Review articles, systematic reviews, and case reports were excluded due to their lower level of scientific evidence. Additionally, articles that addressed patients missing adjacent teeth, or where the lateral incisor was absent due to trauma or dental caries, were not included.

2.3 Information Sources and Search Strategy

Searches were conducted in the following electronic databases in October 2024: 4 WEB OF SCIENCE, SCOPUS, and PUBMED, with the last search occurring on 19/10/24. Search strategies were developed using a process of identifying relevant keywords, phrases, and their combinations to ensure comprehensive coverage of studies aligned with the research objectives.

The keywords used in the advanced search were: "(upper lateral incisor OR maxillary lateral incisor) AND (tooth agenesis OR dental agenesis OR missing tooth OR absent tooth OR congenitally missing) AND 11 (space closure OR space opening OR orthodontic treatment OR dental implant OR single-tooth implant OR 15 fixed partial denture OR maryland dental bridge OR resin-bonded bridge OR invisalign) AND (children OR adolescents OR child OR teenager OR growing patient)." Additionally, reference lists of potential studies were manually reviewed.

2.4 Study Selection Process

Four independent reviewers (P.M., S.C., P.L., F.I.) assessed the quality of the included studies using specified criteria such as selection criteria, methods of outcome evaluation, and data analysis.

This enhanced 'risk of bias' tool additionally includes quality standards for selection, performance, detection, reporting, and other biases. Any differences were settled through conversation or collaboration with other researchers (A.D.I., A.M.I., G.D.). The reviewers screened the records according to the inclusion and exclusion criteria. Doubts have been resolved by consulting the senior reviewer (F.I.). The selected articles were downloaded into Zotero.

2.5 Data Extraction

After thorough reading of the included studies, a list of variables was extracted, providing key information and facilitating a better understanding of the various therapeutic approaches. The variables examined in each study included: author, year of publication, study type, sample size, demographic characteristics of patients (gender and age), type of treatment, and follow-up period.

2.6 Quality Assessment

Each article was evaluated using the Newcastle-Ottawa Scale to assess its rigor and quality. A template of the assessment tool was completed in Microsoft Excel (Windows 11, version 16.59, © 2022 Microsoft) to evaluate the risk of bias.

3. RESULTS

The initial search identified 379 articles, of which 167 were excluded as duplicates. After reviewing the titles and abstracts of the remaining 170 studies, 128 were excluded for being unrelated to the topic or not meeting the eligibility criteria. A total of 42 full-text articles were reviewed, and 35 were excluded for reasons such as studies on adult

patients, loss of lateral incisors due to trauma, inclusion of bilateral [8] agenesis of the upper lateral incisors, reviews, systematic reviews, case reports, and irrelevance to the PICO question or research objectives. Full texts of 5 articles could not be retrieved. Finally, 7 studies were selected [4] for comparison and analysis, and the key results were tabulated (Figure 1).

As outlined in the study by E. Josefsson and R. Lindsten (23), when both [17] treatment options are available, space closure is preferred due to its superior aesthetic and periodontal outcomes. For instance, patients treated with dental implants exhibited issues such as short clinical crowns, discolored gums, and bleeding upon probing. Whenever feasible, space closure should be recommended to enable growing patients to complete their treatment prior to adulthood, thus promoting long-term adaptation [6] of the teeth and supporting structures that appears more natural (23).

Conversely, as discussed in the work by De Marchi et al. (26), both treatment approaches—space closure and space opening—yielded satisfactory [4] functional and periodontal results for patients with congenitally missing maxillary lateral incisors. In their study, bleeding upon probing, probing depths greater than 3 mm, and recession were noted in patients treated by either method [14] as well as in the control group (26).

Furthermore, infraocclusion was a notable issue in patients receiving implants, as reported by A. Jamilian et al. (24). While implant [13] placement has become a common treatment modality, it is important to consider this drawback given that growth has not yet concluded. Additionally, patients who underwent space closure demonstrated better periodontal health compared to those with implants, none of whom exhibited mobility (24).

Is it possible to harness the benefits of dental implants while minimizing or eliminating the complications associated with their placement in growing patients? Authors J. B. Cope and D.M. Fadden (28) attempted to address this question. In their research, they used mini implants as temporary dental implants during adolescence, subsequently replacing them with permanent dental implants after growth had ceased. These authors reported very

positive outcomes in terms of aesthetics and periodontal health, including the absence of infraocclusion and preservation of the bone and soft tissues surrounding the implant during the patient's growth (28).

Similar results were obtained by M. Lacarbonara et al. (25), who also investigated mini implants, regarded as a valid approach to combat the typical bone resorption seen in the premaxilla. They noted that in anterior regions, the dental ridge may be too narrow to accommodate a standard-sized implant. The collected data indicated excellent stability of the mini implant, no progressive peri-implantitis, and satisfactory aesthetic results.

On another note, Mangano et al. (27) focused on the application of Morse taper connection implants, which achieved superior aesthetic and periodontal outcomes compared to conventional implants. Their design enhances the space for connective tissue, thereby improving biological sealing. This increased space facilitates optimal healing of soft tissues, resulting in greater gingival volume and better organization of the peri-implant soft tissues, protecting the bone crest from potential resorption.

Finally, E.W. Amm et al. (29) proposed space closure with canine reshaping as a method for addressing the congenital absence of maxillary lateral incisors in patients exhibiting Class III or Class I skeletal patterns. Utilizing mandibular mini implants for the insertion of Class III elastics, this therapeutic option was shown to be highly effective from both aesthetic and periodontal perspectives.

4.Risk of Bias

TABLE 3. Risk of Bias Assessment

Cohort study

Selection

Representativeness of the Exposed Cohort

Selection of the Non-Exposed Cohort

Ascertainment of Exposure

Demonstration that Outcome of Interest Was Not Present at Start of Study

Comparability

Comparability of Cohorts on the Basis of the Design or Analysis

Outcome

Assessment of Outcome

Was Follow-up Long Enough for Outcomes to Occur?

Adequacy of Follow-up of Cohorts

Case controls

Selection

Is the case definition adequate?

Representativeness of the cases

Selection of Controls

Definition of Controls

Comparability

Comparability of cases and controls on the basis of the design or analysis

Exposure

Ascertainment of exposure

Same method of ascertainment for cases and controls

Non-Response rate

Total score

The risk of bias was evaluated using the Newcastle-Ottawa Scale (Wells et al., 2000), which comprises eight items distributed across three subscales, with a maximum total score of nine. The studies included in this systematic review were categorized as having moderate quality, as they achieved scores of ≥7. An exception is the case series by Cope

and Fadden (28), which exhibited a high risk of bias due to its small sample size.

5. Discussion

When a patient presents with unilateral congenital absence of the maxillary lateral incisor, clinicians face the decision of whether to close the edentulous space. Dentists can approach this issue through various methods. The reviewed literature describes the following treatment options for congenitally absent lateral incisors:

Closing the space through mesial relocation and contouring of the canine;

Opening the space to place a removable or fixed prosthesis or dental implant (26).

Adolescents often feel self-conscious removing their prosthesis and revealing the gap in front of their peers, making removable prosthetics poorly tolerated. Additionally, there is a risk of damaging or breaking the removable prosthesis (28).

Conversely, all fixed prosthetic alternatives necessitate enamel reduction on teeth that are otherwise healthy and do not guarantee optimal dental and periodontal aesthetics. The most common drawback of prosthetic treatment is that it fails to prevent resorption of the alveolar bone and soft tissue contraction in the site of the missing tooth over time. Due to the absence of alveolar tension, prosthetics can lead to the loss of alveolar bone, complicating future dental implant rehabilitation. If an implant and restoration are later chosen, patients often require bone and soft tissue grafts, particularly if they are young at the time of orthodontic treatment (28).

Implant-retained prosthetics offer another treatment avenue for congenitally absent lateral incisors. With a history spanning nearly 50 years, dental implants have established a strong reputation for their high success rates. Jung et al. (31) reported a five-year survival rate of 96.8% for single-tooth implants in a recent comprehensive evaluation (31).

In conclusion, space closure and canine contouring represent a feasible and safe treatment option that yields satisfactory aesthetic and periodontal outcomes. Recent advancements in restorative therapy, including individualized dental bleaching, porcelain

veneers, and hybrid resin reconstructions, demonstrate that combining space closure with aesthetic dentistry can result in highly satisfactory treatment (24).

5.1 Aesthetic Comparison

Achieving aesthetic rehabilitation with dental implants in the anterior region poses one of the greatest challenges in contemporary dentistry. Most implant-supported crowns referenced in the work of Josefsson and Lindsten (23) ultimately fall short over time, a finding echoed in Jamilian et al. (24). One possible explanation is that the alveolar bone in patients continues to grow post-implant treatment, leading to ongoing eruption of neighboring teeth (23). Several studies indicate that craniofacial skeletal changes persist into adulthood, with Iseri and Solow finding that dental eruption continues until approximately 25 years of age (32). In individuals with a high smile line, asymmetric gingival margin levels caused by infraocclusive implant restorations are a clear disadvantage. Consequently, a "gummy smile," or visibility of gingival margins, contraindicates implant replacement for missing upper lateral incisors (24). Conversely, Lacarbonara et al. (25) report no evidence of infraocclusion in patients using mini implants, attributing this to the fact that this type of smaller implant does not integrate into the bone (25).

Moreover, some undesirable aesthetic consequences of implant-supported crowns, such as gingival discoloration, have been noted by Jamilian et al. (24). The gingiva surrounding implants appears significantly worse compared to that of patients treated with space closure. This finding aligns with the work of Dueled et al. (33), who observed mucosal discoloration in most patients with implant-supported crowns. The resorption of alveolar bone beneath the mucosa is the cause of this discoloration (23). Several studies have indicated that the height and width of the bony crest are reduced following orthodontic space opening for implant placement. Various methods have been proposed to address the issue of insufficient bone thickness in cases requiring implant-prosthetic rehabilitation, including techniques to alter the implant insertion axis, which may expose the prosthetic

restoration to a specific risk of failure, ridge augmentation (guided bone regeneration) using resorbable membranes, and bone grafts. These procedures elevate the risk of potential adverse effects, as well as increase costs and treatment duration (25). Regarding color, all implant-supported crowns in Josefsson and Lindsten's study (23) were found to have ideal or acceptable color, which was superior to the canine color used to replace the missing maxillary lateral incisor (23). This conclusion is also supported by other studies, such as that of De Marchi et al. (26), which highlights the low adhesion of bacterial plaque to porcelain implants compared to composite resin, which tends to promote greater accumulation of debris and pigmentation due to difficulties achieving adequate polishing. The authors emphasize that a long-term reevaluation of the studied sample or a larger sample size could better demonstrate the behavior of both treatment types (26).

5.2 Comparison Based on Periodontal Parameters

In the study by Josefsson and Lindsten (23), no differences in bleeding on probing were found among patient groups (23). Chang et al. (34) compared the periodontal status of dental implants to contralateral natural teeth in a follow-up study and discovered greater bleeding upon probing around the implant, along with increased plaque index and probing depth (34). Conversely, Jamilian et al. (24) reported that patients treated with space opening exhibited more bleeding than those treated with space closure. Furthermore, most implants showed an increase in probing depth exceeding 3 mm, whereas only a few teeth in the space closure group displayed an increase beyond this threshold (24). De Marchi et al. (26) confirmed these findings, noting probing depths exceeding 3 mm in the space closure group due to the mesial movement of canines (26). Additionally, the same article indicates that more implant-supported crowns exhibited gingival recession than recontoured canines. Literature indicates that gingival recession of 1 mm or more occurs in over half of the population, regardless of oral hygiene level, and increases with age; its etiology relates to anatomical, physiological, and pathological variables (35). One common objection to placing implants in the premaxilla is the unaesthetic appearance

of the soft tissue surrounding the implant. The bone level around the neck of the implant must be preserved to achieve optimal aesthetic results. However, when a post is attached to a dental implant at the crestal level, bone loss around the implant almost invariably occurs. Surgical trauma or micro-movements of the post are believed to contribute to early crestal bone loss (27). The reviewed literature indicates an increase in mobility among patients treated with implants, while none of the patients in the space closure group exhibited dental movement (24). Morse implants are designed to mitigate micro-movements at the implant-post junction, addressing one of the causes of crestal bone loss around implants as previously described. Furthermore, a more adequate volume of peri-implant soft tissues protects the bony crest from resorption. Therefore, these implants can facilitate optimal recovery of soft tissues (27).

5.3 Advantages and Disadvantages Comparison

After analyzing the selected studies and their references, the following are the main advantages of space closure with canine replacement compared to implants. First, the treatment is completed immediately after orthodontics, which is crucial for adolescent patients. Additionally, because the tooth has migrated alongside its bone and surrounding tissues, there is a lower risk of periodontal diseases with space closure. Lastly, due to the absence of bone loss and infraocclusion, space closure provides generally favorable aesthetic outcomes for patients (24).

However, notable disadvantages include that porcelain is inherently a more aesthetic material than composite. Following treatment, further restorative therapy or whitening may be necessary if the canine appears excessively dark, and maintaining long-term retention results can be challenging. Implant replacement seems to present more disadvantages than advantages. The primary drawback of implants is osteointegration, which carries the risk of infraocclusion. This issue may be addressed through the use of mini implants, which, being smaller and not requiring integration into the bone, allow for the surrounding bone tissue to grow along with adjacent teeth. Periodontal issues, such as the typical

migration of gum and bone with traditional implants, can be managed using Morse taper connection implants, whose design enhances biological sealing to ensure excellent soft tissue healing, resulting in increased gingival volume and better organization of peri-implant soft tissues, while also protecting the bony crest from resorption (27).

As previously mentioned, a significant disadvantage of the implant alternative is that adolescents must wait several years after orthodontic treatment before implant placement. During this interim period, patients often rely on temporary restorations that can present numerous issues and may frequently require replacement. Finally, opening the space and placing an implant necessitates perfect teamwork among the orthodontist, oral surgeon, and prosthodontist, or the result may be compromised (24). Furthermore, given that craniofacial skeletal changes continue throughout adolescence and early adulthood, implant rehabilitation should be completed at the conclusion of craniofacial development (26). The infraocclusion of conventional implants, due to the eruption of adjacent teeth, marginal bone loss around these teeth, and potential vertical angular defects between the implant and neighboring teeth, poses challenges for osteointegrated dental implants in growing adolescents (28).

All patients with implants in the study by Josefsson and Lindsten (23) exhibited infraocclusion exceeding 1 mm. The implants in this study were standard implants and had undergone osteointegration. This context prompted Lacarbonara et al. (25) to suggest that larger implants should be avoided in young patients to minimize the risk of infraocclusion (25). Hence, it is evident that space closure with canine contouring remains a highly recommended treatment option for the management of congenital absence of lateral incisors.

5.5 Limitations

When comparing the results of various studies, it is crucial to acknowledge the limitations inherent in the analyzed research.

In the study by E. Josefsson and R. Lindsten (23), a primary limitation is the small sample

size and the fact that examinations were conducted by a single examiner. Additionally, in the research by A. Jamilian et al. (24), patients were assigned to implant or orthodontic space closure (OSC) groups based on the inter-dental spacing, which raises concerns about the lack of randomization in group allocation. Despite this, the findings from this study should be considered when planning treatment for patients in borderline cases where treatment options may include implants or orthodontic space closure, as it thoroughly outlines the advantages and disadvantages of each approach.

The retrospective design of the studies by M. Lacarbonara et al. (25) and Mangano et al. (27) also presents limitations. Although retrospective studies can yield results relatively quickly since the necessary time for outcome assessment has already passed, they generally provide less reliable results compared to prospective studies, such as the research conducted by E.W. Amm et al. (29). Case series, like those presented by J.B. Cope and D.M. Fadden (28), also carry a higher risk of bias, highlighting the need for well-structured cohort studies in the future to validate their findings.

Furthermore, as pointed out by De Marchi et al. (26), a long-term reevaluation of the studied sample or the inclusion of a larger sample size is essential to better demonstrate the outcomes associated with both treatment types. To achieve the optimal treatment option for each patient, these data should be analyzed in conjunction with the expectations of the patients and their parents, as well as the professional team's experience.

This systematic review has several weaknesses, including a high level of bias in the study by J.B. Cope and D.M. Fadden (28), the absence of gray literature review, small sample sizes, and short follow-up periods. Additionally, more prospective cohort studies are needed to provide stronger scientific evidence in the future.

6. Conclusion

Based on the findings from the reviewed articles, the following conclusions can be drawn:

The decision to either open or close the space resulting from agenesis should consider the

type of malocclusion, the anterior dental relationship, the available space, and the condition of adjacent teeth.

In Class II Division 1 cases, particularly when there is crowding in the lower arch that necessitates extractions, [14] as well as in Class I cases with lower arch crowding, increased overbite, and anterior open bite, the trend leans toward space closure. Conversely, in Class III or Class I cases with a tendency toward Class III, decreased overbite, and deep bite, [1] it is recommended to open the space for the agenesis incisor and replace it with an implant.

The limitations associated with the option of space opening and replacement with an implant in growing patients relate to the appropriate age for implant placement. However, the current use of mini implants and [7] Morse taper connection implants significantly reduce many of the aesthetic and periodontal challenges commonly associated with conventional implants

Sources

1	https://pmc.ncbi.nlm.nih.gov/articles/PMC9172892/<br>INTERNET	2%
2	https://www.mdpi.com/2227-9067/11/11/1375/xml<br>INTERNET	1%
3	https://progressinorthodontics.springeropen.com/articles/10.1186/s40510-015-0072-2<br>INTERNET	1%
4	https://pubmed.ncbi.nlm.nih.gov/27476355/<br>INTERNET	1%
5	https://decisionsindentistry.com/article/diagnosing-developmental-tooth-anomalies/<br>INTERNET	1%
6	https://pmc.ncbi.nlm.nih.gov/articles/PMC3844689/<br>INTERNET	1%
7	https://www.academia.edu/52397441/Morse_taper_connection_implants_supporting_planned_maxillary_and_mandibular_bar_retained_overdentures_a_5_year_prospective_multicenter_study<br>INTERNET	1%
8	https://www.ncbi.nlm.nih.gov/pmc/articles/PMC10290303/<br>INTERNET	<1%
9	https://www.ncbi.nlm.nih.gov/books/NBK592390/<br>INTERNET	<1%
10	https://www.dovepress.com/get_supplementary_file.php?f=132940_1.pdf<br>INTERNET	<1%
11	https://pmc.ncbi.nlm.nih.gov/articles/PMC10461125/<br>INTERNET	<1%
12	https://www.nature.com/articles/s41415-023-6384-6<br>INTERNET	<1%
13	https://pmc.ncbi.nlm.nih.gov/articles/PMC10528222/<br>INTERNET	<1%
14	https://pmc.ncbi.nlm.nih.gov/articles/PMC9931762/<br>INTERNET	<1%

15 https://www.ncbi.nlm.nih.gov/pmc/articles/PMC3258188/
INTERNET
<1%

16 https://pmc.ncbi.nlm.nih.gov/articles/PMC7579235/
INTERNET
<1%

17 https://www.ncbi.nlm.nih.gov/books/NBK573071/
INTERNET
<1%

18 https://www.researchgate.net/publication/323499191_Long-term_effects_of_traumatic_dental_injuries_of_primary_dentition_on_permanent_successors_A_retrospective_study_of_596_teeth
INTERNET
<1%

19 https://www.nature.com/articles/s41598-020-58565-8
INTERNET
<1%

20 https://pmc.ncbi.nlm.nih.gov/articles/PMC6337530/
INTERNET
<1%

21 https://pmc.ncbi.nlm.nih.gov/articles/PMC5952365/
INTERNET
<1%

22 https://www.ncbi.nlm.nih.gov/pmc/articles/PMC8585468/
INTERNET
<1%

23 https://pmc.ncbi.nlm.nih.gov/articles/PMC8388057/
INTERNET
<1%

24 https://opendentistryjournal.com/VOLUME/8/PAGE/289/FULLTEXT/
INTERNET
<1%

25 https://pubmed.ncbi.nlm.nih.gov/34564740/
INTERNET
<1%

26 https://pubmed.ncbi.nlm.nih.gov/12636127/
INTERNET
<1%

27 https://www.researchgate.net/figure/Cervical-vertebrae-C2-C3-and-C4-revealed-on-lateral-cephalometric-radiograph-for_fig2_5406014
INTERNET
<1%

28 https://systematicreviewsjournal.biomedcentral.com/articles/10.1186/s13643-021-01626-4
INTERNET
<1%

29	https://link.springer.com/chapter/10.1007/978-3-031-23980-9_2<br>INTERNET<br><1%
30	https://www.nidcr.nih.gov/health-info/ectodermal-dysplasia#:~:text=Ectodermal dysplasias (ED) are disorders that affect the,specific types of this condition have been identified.<br>INTERNET<br><1%
31	https://training.cochrane.org/handbook/current/chapter-25<br>INTERNET<br><1%
32	https://www.sciencedirect.com/science/article/pii/S1013905224001780<br>INTERNET<br><1%
33	https://onlinelibrary.wiley.com/doi/full/10.1111/prd.12613<br>INTERNET<br><1%

EXCLUDE CUSTOM MATCHES ON

EXCLUDE QUOTES ON

EXCLUDE BIBLIOGRAPHY ON
